# The Effects of Disinfection Byproduct 2,6-Dichloro-1,4-benzoquinone on the Cyanobacterium *Microcystis aeruginosa*: From the Perspectives of Biochemistry and Non-Targeted Metabolomics

**DOI:** 10.3390/toxics13010064

**Published:** 2025-01-17

**Authors:** Tianqi Zhang, Zhaoyang Wang, Liang Wu, Chaonan Liu, Liang Meng, Fuxiang Tian, Meifang Hou, Haizhuan Lin, Jing Ye

**Affiliations:** 1 School of Chemical and Environmental Engineering, Shanghai Institute of Technology, Shanghai 201418, China; 236061257@mail.sit.edu.cn (T.Z.); 15850606769@163.com (Z.W.); lcn@sit.edu.cn (C.L.); 13916841275@163.com (F.T.); mfhou@sit.edu.cn (M.H.); 2 Department of Chemical and Environmental Engineering, University of California, Riverside, CA 92521, USA; chriswu1009@hotmail.com; 3 School of Environmental and Geographical Sciences, Shanghai Normal University, Shanghai 200234, China; mengliang@shnu.edu.cn; 4 Yangtze River Delta Urban Wetland Ecosystem National Field Scientific Observation and Research Station, Shanghai 201722, China; 5 College of Architecture and Energy Engineering, Wenzhou University of Technology, Wenzhou 325000, China; 6 State Key Laboratory of Environmental Criteria and Risk Assessment, Chinese Research Academy of Environmental Sciences, Beijing 100012, China

**Keywords:** 2,6-dichloro-1,4-benzoquinone, *Microcystis aeruginosa*, toxic effect, non-targeted metabolomics, water quality criteria

## Abstract

2,6-Dichloro-1,4-benzoquinone (2,6-DCBQ) is an emerging chlorinated disinfection byproduct (DBP) in bodies of water. However, this compound poses an unknown toxic effect on cyanobacteria. In this study, the toxicological mechanisms of 2,6-DCBQ in *Microcystis aeruginosa* (*M. aeruginosa*) were investigated through physiological and nontargeted metabolomic assessments. The results show that 2,6-DCBQ inhibited the growth of *M. aeruginosa*, reduced its photosynthetic pigment and protein contents, increased the levels of reactive oxygen species, damaged the antioxidant defense system, and aggravated the cytomembrane. Meanwhile, 2,6-DCBQ stimulated the production and release of microcystin-LR (MC-LR) and altered the transcripts of genes associated with its synthesis (*mcyA*, *mcyD*) and transport (*mcyH*). In addition, nontargeted metabolomics of *M. aeruginosa* cells exposed to 0.1 mg/L 2,6-DCBQ identified 208 differential metabolites belonging to 10 metabolic pathways and revealed the considerable interference caused by 2,6-DCBQ among ABC transporters, the two-component system, and folate biosynthesis. This study deepens the understanding of the physiological and nontargeted metabolomic responses of *M. aeruginosa* exposed to 2,6-DCBQ, offers insights into the toxic effect of 2,6-DCBQ on *M. aeruginosa*, and provides a theoretical basis for the ecological risk assessment of emerging DBPs in accordance with water quality criteria.

## 1. Introduction

The COVID-19 pandemic spread globally and greatly threatened human health [1]. The worldwide COVID-19 pandemic has resulted in a substantial rise in the use of disinfectants around the globe. Excessive use of disinfectants leads to increased concentrations of residual chlorine and disinfection byproducts (DBPs) in water bodies [2]. Thus far, DBPs have been extensively detected in a variety of reclaimed and surface waters [3]. Although the environmental levels of DBPs (~µg/L) are typically considerably lower than their effective toxic concentrations (~mg/L), recent studies have shown that DBPs at environmentally relevant concentrations can also pose environmental risks [4,5]. At present, over 900 DBPs have been detected [6], but only a limited number have been investigated.

Halobenzoquinones (HBQs), a new class of DBPs [7,8], have garnered research interest due to studies demonstrating their potential cytotoxicity, genotoxicity, and carcinogenicity [9,10]. Toxicological experiments have confirmed the high cytotoxicity of HBQs, with toxicity levels approximately 10–1000 times higher than those of regulated DBPs, such as trichloromethane and halogenated acetic acids [7]. 2,6-Dichloro-1,4-benzoquinone (2,6-DCBQ) is the most frequently detected type of HBQs, with the highest detection concentration of 274.5 ± 13.0 ng/L [11,12]. The toxic effect of 2,6-DCBQ is an important issue in the research on DBPs. Previous research has indicated that 2,6-DCBQ-induced toxicity is mainly due to the presence of reactive oxygen species (ROS) [13]. However, the toxicity and potential mechanisms of 2,6-DCBQ have not been explored, especially in aquatic ecosystems.

Cyanobacterial blooms have attracted worldwide attention in the past several decades [14]. Cyanobacterial blooms have caused detrimental effects on the ecological safety of water environments, including the restriction of light penetration, the depletion of dissolved oxygen, and the mortality of aquatic organisms [15,16,17]. In addition, eutrophic lakes have encountered new challenges due to the introduction of emerging contaminants [18,19]. *Microcystis aeruginosa* is the most common type of phytoplankton [20,21]. *M. aeruginosa*, as a primary producer in aquatic habitats, is a major contributor to global biogeochemical cycles [22]. However, as a potential toxic cyanobacterium, it produces microcystins, which exhibit significant toxicity to other aquatic organisms. Cyanobacteria that have been exposed to environmental stressors experience physiological changes, which can be used in the effective identification of the toxic effects of pollutants and the further unveiling of their underlying mechanisms. The emerging contaminants (such as DBPs) released into the aquatic environment may contribute to the outbreak of cyanobacterial blooms through their effects on cyanobacterial growth and metabolism [23,24]. Therefore, conducting studies on the impact of emerging contaminants, such as 2,6-DCBQ, on *M. aeruginosa* has profound significance.

This study investigated the physiological response of *M. aeruginosa* exposed to 2,6-DCBQ. The cell density and contents of chlorophyll (Chl)-a, carotenoids, adenosine triphosphate (ATP), proteins, and glucose were examined. Measurements were conducted for the ROS level, microcystin-LR (MC-LR) content, lipid peroxidation, and activities of antioxidant-related enzymes in *M. aeruginosa*. In addition, observations were focused on the morphology and ultrastructural characteristics of cyanobacteria cells. Finally, nontargeted metabolomics was applied in the metabolite analysis and identification of toxicity biomarkers. The results can aid in the elucidation of the toxicity mechanisms of 2,6-DCBQ on *M. aeruginosa* and provide a theoretical basis for the ecological risk assessment of emerging DBPs in accordance with water quality criteria.

## 2. Materials and Methods

### 2.1. Chemicals and M. aeruginosa Cultivation

2,6-Dichloro-1,4-benzoquinone (2,6-DCBQ) (CAS No. 697-91-6) was obtained from TCL Co., Ltd. (Shanghai, China). An MC-LR standard sample (purity ≥ 95%) was obtained from Puhuaren Biological Development Co., Ltd. (Beijing, China).

*M. aeruginosa* (FACHB-905) was acquired from the Freshwater Algae Culture collection at the Institute of Hydrobiology, Chinese Academy of Sciences (Wuhan, China). The cultivation conditions were referenced from our previous paper [25]. Based on the preliminary experiments, exposure concentrations of 2,6-DCBQ were set as follows: 0.01, 0.05, 0.1, 0.2, and 0.5 mg/L. According to the OECD Test Guideline No. 201, dimethyl sulfoxide (purity ≥ 99.9%) was used as a carrier to dissolve 2,6-DCBQ, which was added to the *M. aeruginosa* culture media at a volume below 0.01% (*v*/*v*).

### 2.2. Measurements of Growth and the Contents of Chl-a, Carotenoid, and Protein

Based on preliminary experiments, five concentrations (0.01, 0.05, 0.1, 0.2, and 0.5 mg/L) were selected for testing. Guava flow cytometry (Luminex, Austin, TX, USA) was used to acquire the standard curve correlating the cell density with the optical density. The measurements were analyzed using the following equations [25]:

Cell density = 13.77488 × OD680 + 0.75405 (R^2^ = 0.9934) (1)

We determined the content of chlorophyll a and carotenoids in the *M. aeruginosa* using the N, N-dimethylformamide method. Each group of *M. aeruginosa* cells were collected on days 3, 6, and 9 for the chl-a and carotenoid content measurements. The measurements were analyzed using the following equations:

Chl-a = 13.7 × OD665 − 5.76 × OD649(2)

Carotenoid = (1000 × OD470 − 1.91chl-a)/225(3)

The intracellular protein content was measured according to the instructions of the kit (A045-2-2, Jiancheng Bioengineering Institute, Nanjing, China) on days 3 and 6, which are based on the basic principles of the Bradford method.

### 2.3. ATP Content and Glucose Content Detection

The ATP content was determined according to phosphomolybdic acid colorimetry. The ATP content was measured according to the instructions of the kit (A095-1-1, Jiancheng Bioengineering Institute, Nanjing, China) on days 3 and 6. The luminescence intensity was measured at 636 nm. The glucose content was determined according to the glucose oxidase method. The glucose content was measured according to the instructions of the kit (F006-1-1, Jiancheng Bioengineering Institute, Nanjing, China) on days 3 and 6.

### 2.4. Determination of Oxidative Stress Biomarkers

After 24 and 48 h of exposure, the ROS level was measured according to the instructions of the kit (A106-1-2), which are based on the basic principles of 2′,7′-Dichlorodihydrofluorescein diacetate. After 24 and 48 h of exposure, the lipid hydroperoxide (LPO) content was measured according to the instructions of the kit (A106-1-2), which are based on the basic principles of the colorimetric method. The catalase (CAT) and superoxide dismutase (SOD) activities were determined according to the ammonium molybdate method and a xanthine oxidase assay, respectively. The CAT and SOD activities were measured according to the instructions of the kits (A007-1-1 and A001-3-2). The operation and calculation procedures were conducted in accordance with the instructions provided by the kit (Jiancheng Bioengineering Institute, Nanjing, China).

### 2.5. Scanning Electron Microscope (SEM) and Transmission Electron Microscope (TEM) Observations

SEM and TEM observations were conducted according to our previous studies [25]. After 72 h of exposure, 1mL of the cell sample was centrifuged at 4000× *g* (4 °C, 8 min), and the precipitated cells were collected. The sediment was suspended in 1.5mL PBS. The samples were fixed in 25% glutaraldehyde solution at 4 °C in the dark for 12 h, and washed three times with 0.1 M PBS (pH = 7.0). Then, the solution was dehydrated in different concentration gradients of ethanol for 15 min each and treated with 100% ethanol for 20 min. The samples were soaked in 100% propylene oxide for 20 min, then treated with a mixture of embedding agent and acetone, and embedded in epoxy resin. The surface morphology of the cells was observed using SEM at 3.0 kV with an SU8010 instrument (HITACHI, SU8010, Hitachi, Ltd., Tokyo, Japan), while the ultrastructure of the cells was observed using TEM (HITACHI H-7650, Hitachi, Ltd., Tokyo, Japan).

### 2.6. Detection of Microcystin-LR in M. aeruginosa

The MC-LR content was measured according to our previous studies [25]. After 72 and 144 h of exposure, 40 mL of culture media was centrifuged at 4000× *g* (4 °C, 8 min). The supernatant was collected as extracellular MC-LR. The pellet was utilized as an intracellular solution for MC-LR extraction. The precipitate was dissolved in 10 mL of 50% methanol and subjected to sonication at 40% power using an ultrasonic cell disruptor in an ice-water bath (below 4 °C) for 8 min. The supernatant obtained after centrifugation at 7000× *g* (4 °C, 10 min) was used for further measurement of the intracellular MC-LR.

The samples gathered underwent solid-phase extraction (SPE) and analyzed by HPLC (Shimadzu, TC-20A, Shanghai, China) [26]. The SPE cartridge used contained C18 silica gel (TS208-001, Shanghai Titan Technology Co., Ltd., Shanghai, China), with a packing material of 500 mg/6 mL per cartridge. The extraction process was divided into four steps: activation (10 mL of pure methanol and 10 mL of distilled water), sample injection, leaching (10 mL of 20% methanol), and elution (5mL of methanol). After the SPE process, the resulting eluate was dried using nitrogen at a temperature of 40 °C until it was almost dry and resuspended in 1 mL of chromatography-grade methanol. The eluate was then stored at a temperature of −20 °C for analysis. The samples were measured by HPLC (UV detector, 238 nm). The mobile phase consisted of a mixture of water and methanol at a volumetric ratio of 40:60 (*v*:*v*). The injection volume was 10 μL, with a flow rate of 1.0 mL/min. The column temperature was maintained at 35 °C, and each sample was analyzed within 15 min. The limit of determination of MC-LR is 100 μg/L.

### 2.7. Extraction of Metabolites and Nontargeted Metabolomics Analysis

Extraction and analysis of the metabolites were conducted by Shanghai Luming Biotechnology Co., Ltd. (Shanghai, China). To analyze the metabolites, 2 mL of the cultural samples from the CON (control group) and the 0.1 mg/L group on day 9 were collected and centrifuged at 100× *g* for 5 min [27,28]. The pellets were gently mixed and washed with 1 mL of PBS, and the cells of *M. aeruginosa* were immediately frozen in liquid nitrogen for further analysis. Then, the samples were analyzed through ultra-performance liquid chromatography–mass spectrometry (ACQUITY UPLC-MS, Waters Corporation, Milford, MA, USA) equipped with the ACQUITY UPLC BEN C18 column (100 mm × 2.1 mm, 1.7 μm; Waters Corporation). The mobile phases consisted of 0.1% formic acid in water/acetonitrile (95/5, *v*/*v*, solvent A) and 0.1% formic acid in acetonitrile/isopropanol/water (47.5/47.5/5, *v*/*v*, solvent B). The sample injection volume was 5 μL, and the flow rate was set to 0.4 mL/min. The column temperature was maintained at 45 °C.

Metabolites were identified on the metabolome database. The online platform OECloud tools (https://cloud.oebiotech.com, accessed on 14 January 2025) was used to summarize and analyze the data in bioinformatics, accessed on 5 March 2024. The roles of these metabolites and metabolic pathways were analyzed using the KEGG database.

### 2.8. Quantitative Real-Time PCR Analysis

Quantitative real-time PCR analysis was conducted according to our previous studies [25]. The fold change in gene expression was determined using the 2^−ΔΔCt^ method. Cultural samples from the CON and the 0.1 mg/L 2,6-DCBQ treatment were collected on day 3. The primers of the target genes (*mcyA*, *mcyD*, *mcyH*, *rbcL*, *ftsH*, *recA*, *bchL*, *pckA*, *pgk*, and *psaB*) are listed in Table S1.

### 2.9. Reproducibility of the Results and Statistical Analysis

According to the OECD Test Guideline No. 201, we conducted 3 independent experiments, each with 3 replicates. Statistical analysis was performed using GraphPad Prism 8.0.1 (GraphPad Software, San Diego, CA, USA) and SPSS 27.0 (SPSS Inc., Chicago, IL, USA) to determine the significance among the treatments. One-way analysis of variance (ANOVA) was used to determine the differences between the control and treatment groups, and *p* < 0.05 was considered statistically significant. In addition, the statistical methods for nontargeted metabolomics included principal component analysis (PCA), partial least squares discriminant analysis (OPLS-DA), metabolic pathway enrichment analysis, and heatmap clustering analysis.

## 3. Results

### 3.1. Physiological Responses of M. aeruginosa Exposed to 2,6-DCBQ

#### 3.1.1. Inhibitions of Growth, Photosynthetic Pigments, and Protein Contents

**Figure 1:**
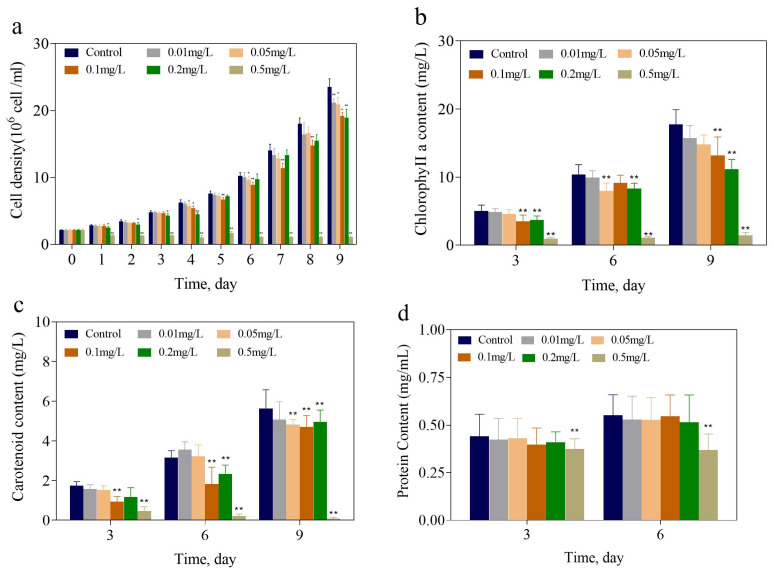
Cell density (**a**) of *M. aeruginosa* treated with 0.01, 0.05, 0.1, 0.2, and 0.5 mg/L of 2,6-DCBQ for 9 days. Concentrations of chlorophyll a (**b**) and carotenoids (**c**) of *M. aeruginosa* treated with mg/L 2,6-DCBQ on days 3, 6, and 9. Protein content (**d**) of *M. aeruginosa* treated with mg/L 2,6-DCBQ on days 3 and 6. Results are presented as means ± standard deviations. * Indicates *p* < 0.05; ** indicates *p* < 0.01 relative to the control by ANOVA.

Figure 1a shows the response of growing *M. aeruginosa* to various concentrations of 2,6-DCBQ. High concentrations of 2,6-DCBQ (0.2 and 0.5 mg/L) significantly inhibited the growth of *M. aeruginosa*. Additionally, 0.05 and 0.1 mg/L 2,6-DCBQ also exhibited significant inhibition effects on day 4 (*p* < 0.05). With increasing concentration and exposure time, concentration- and time-dependent effects were observed.

Growth inhibition can be indicated by the variation in the content of photosynthetic pigments. The 2,6-DCBQ group contained considerably decreased chl-a levels after 3 and 6 days of treatment (Figure 1b). On the 9th day, 0.1, 0.2, and 0.5 mg/L 2,6-DCBQ substantially inhibited chl-a, with inhibition rates of 25.17%, 36.57%, and 92.18%, respectively. The results indicate the inhibited or disrupted photosynthetic system of *M. aeruginosa*, which in turn possibly affected its growth and proliferation. In addition, the content of carotenoids significantly decreased during exposure to 0.1 and 0.5 mg/L 2,6-DCBQ on day 3, with inhibition rates of 47.06% and 72.94%, respectively (Figure 1c). In addition, 0.5 mg/L 2,6-DCBQ resulted in substantial inhibitions in the total soluble protein content, with inhibition rates of 15.9% and 35.7% on days 3 and 6, respectively (Figure 1d).

#### 3.1.2. ATP and Glucose Contents

**Figure 2:**
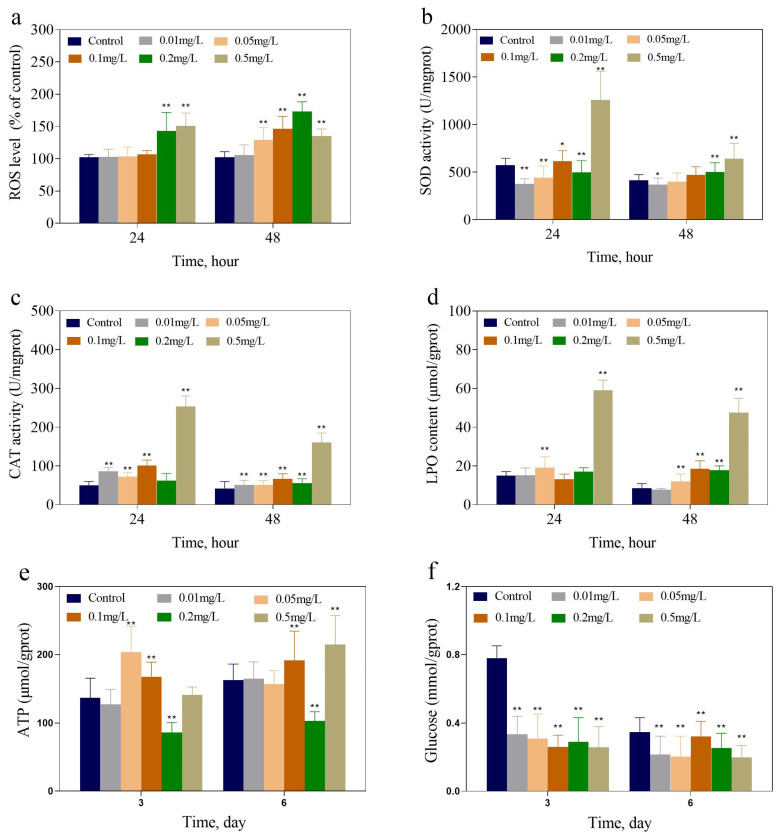
ROS level (**a**), SOD activity (**b**), CAT activity (**c**), and LPO content (**d**) of *M. aeruginosa* treated with 2,6-DCBQ on 24 and 48 h. ATP (**e**) and glucose (**f**) of *M. aeruginosa* treated with 2,6-DCBQ on days 3 and 6. Results are presented as means ± standard deviations. * Indicates *p* < 0.05; ** indicates *p* < 0.01 relative to the control by ANOVA.

Figure 2e,f display the ATP and glucose contents of *M. aeruginosa* exposed to 2,6-DCBQ on days 3 and 6, respectively. The contents of ATP decreased when *M. aeruginosa* was exposed to 0.2 mg/L 2,6-DCBQ for 3 days. In addition, the ATP content significantly increased (*p* < 0.01) when *M. aeruginosa* was exposed to 0.1 and 0.5 mg/L 2,6-DCBQ for 3 and 6 days. The glucose content considerably declined with the increase in 2,6-DCBQ concentrations.

#### 3.1.3. ROS Level, LPO Content, and Detection of Antioxidant Activities

The antioxidant activities were evaluated to investigate the oxidative stress induced by 2,6-DCBQ in *M. aeruginosa*. The intracellular ROS level of *M. aeruginosa* increased significantly during exposure to 0.1 and 0.2 mg/L 2,6-DCBQ (Figure 2a).

Figure 2b,c show the activities of superoxide dismutase (SOD) and catalase (CAT) in *M. aeruginosa* exposed to 2,6-DCBQ. The activities of SOD and CAT presented concentration-related effects. The 0.5 mg/L 2,6-DCBQ increased the SOD activity on days 1 and 2. During *M. aeruginosa* exposure to 0.01, 0.05, 0.1, 0.2, and 0.5 mg/L 2,6-DCBQ, CAT activities were substantially increased by 21.9%, 22.1%, 58.5%, 34.1%, and 290% of the control on day 2 (Figure 2c), respectively. Similarly, the LPO content revealed a significant increase in *M. aeruginosa* when exposed to 0.5 mg/L of 2,6-DCBQ (Figure 2d).

### 3.2. Cell Morphology and Ultrastructure Characteristics

**Figure 3:**
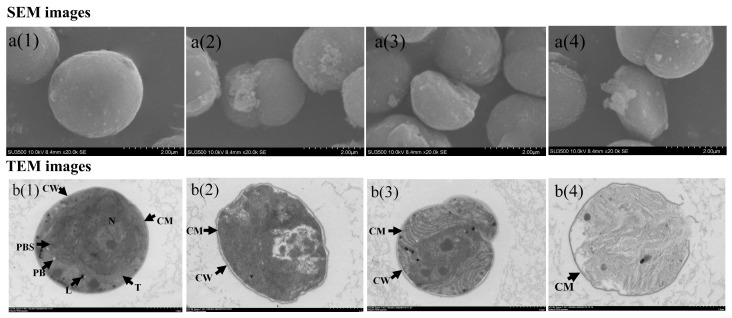
The effects of the CON (**a(1)**,**b(1)**) and 0.1 mg/L of 2,6-DCBQ on the ultrastructure (TEM, (**b(2)**–**b(4)**)) and surface morphology (SEM, (**a(2)**–**a(4)**)) of *M. aeruginosa* cells after 3 days of exposure. The components pictured include the nucleoid (N), phycobilisome (PBS), polyphosphate bodies (PB), thylakoids (T), leucoplast (L), cell wall (CW), and cell membrane (CM).

Figure 3a(1) shows that the surface structure of healthy *M. aeruginosa* cells was smooth and plump, without obvious damage or rupture. The cell membrane (CM) and cell wall (CW) were intact, and the cells exhibited round or oval shapes. However, after the treatment with 0.1 mg/L 2,6-DCBQ, the cells exhibited a shriveled surface morphology, deviating from their normal spherical shape, with a wrinkled and rough surface. The SEM images reveal the presence of abundant extracellular mucus surrounding the cells (Figure 3a(2)). The TEM images show that the CM and CW were rough and fragmented. The thylakoid of *M. aeruginosa* was dispersed (Figure 3b(2),b(3)). These results indicate that 2,6-DCBQ exerted acute toxicity effects on *M. aeruginosa* by inhibiting cell growth and photosynthesis. The disruption of CM further led to the release of intracellular substances (such as MCs) into the aquatic environment, accelerating the death of cells and polluting the water environment.

### 3.3. MC-LR Content

**Figure 4:**
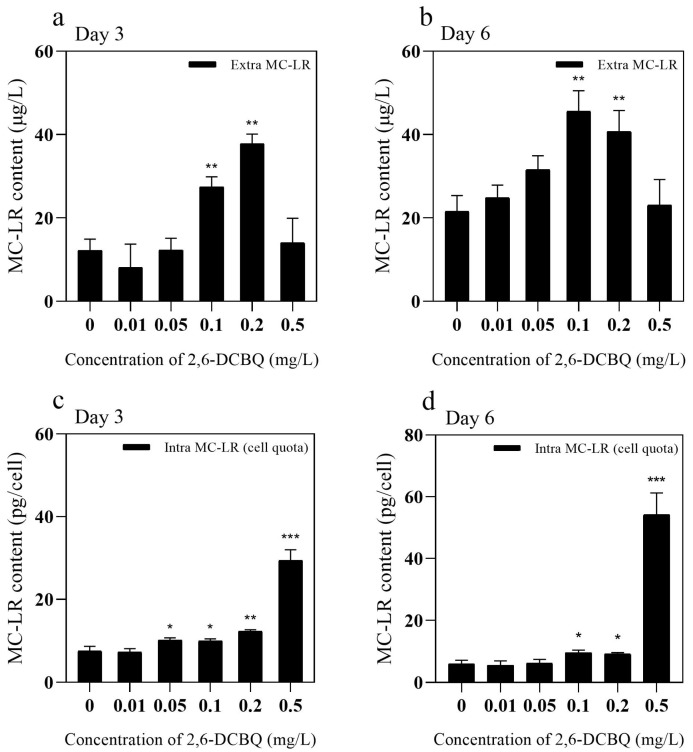
Effects of 2,6-DCBQ on the contents of extracellular (**a**,**b**) and intracellular (cell quota) (**c**,**d**) microcystin-LR in *M. aeruginosa*. Results are presented as means ± standard deviations. * Indicates *p* < 0.05; ** indicates *p* < 0.01; *** indicates *p* < 0.001 relative to the control by ANOVA.

Figure 4 indicates that 0.1 and 0.2 mg/L 2,6-DCBQ stimulated the synthesis of MC-LR (*p* < 0.01). On days 3 and 6, the content of intracellular MC-LR increased when *M. aeruginosa* was exposed to 0.1 and 0.2 mg/L 2,6-DCBQ. Similarly, the extracellular MC-LR contents were increased when exposed to 0.1 and 0.2 mg/L 2,6-DCBQ. The results indicate that 2,6-DCBQ stimulated the production and release of MC-LR in *M. aeruginosa*.

### 3.4. Nontarget Metabolomics Analysis

#### 3.4.1. Metabolite Detection and Analysis

**Figure 5:**
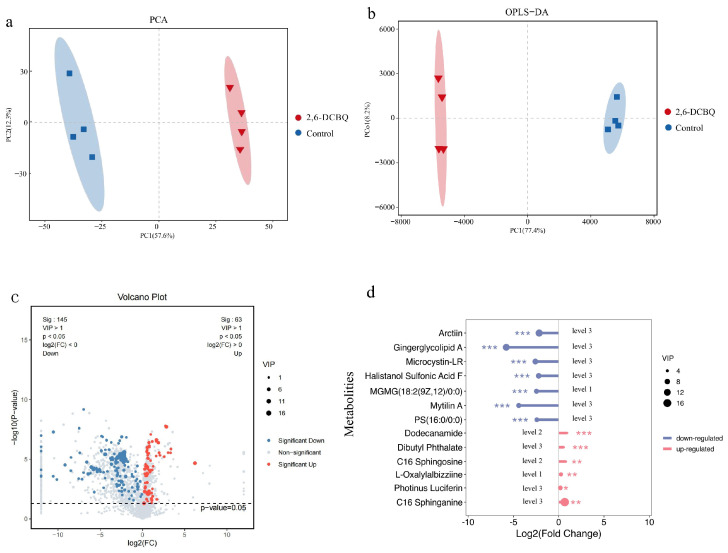
Two-dimensional scores plot of principal component analysis (PCA) (**a**). Two-dimensional scores plot of orthogonal partial least squares discriminant analysis (OPLS-DA) (**b**). Volcano plot of different metabolites (**c**). Lollipop map of main differential metabolites (**d**). The level indicates the accuracy of the metabolite identification, with smaller level numbers representing higher accuracy. * Indicates *p* < 0.05; ** indicates *p* < 0.01; *** indicates *p* < 0.001 relative to the control by *t* test.

We analyzed the changes in the metabolites in *M. aeruginosa* after exposure to 2,6-DCBQ. Figure 5a,b shows the score plots of principal component analysis (PCA) and orthogonal partial least squares discriminant analysis (OPLS-DA), including those of the quality control samples. The scoring plots, which are based on positive ion patterns, demonstrate the overall differences between the sample groups and the variability within each group. As shown in Figure 5a, PCA revealed distinct distributions within each group and effectively distinguished the two sample groups. The consistency of the results from the PLS-DA further supports these findings (Figure 5b).

#### 3.4.2. Identification of Differential Metabolites (DMs) and Metabolic Pathway Analysis

**Figure 6:**
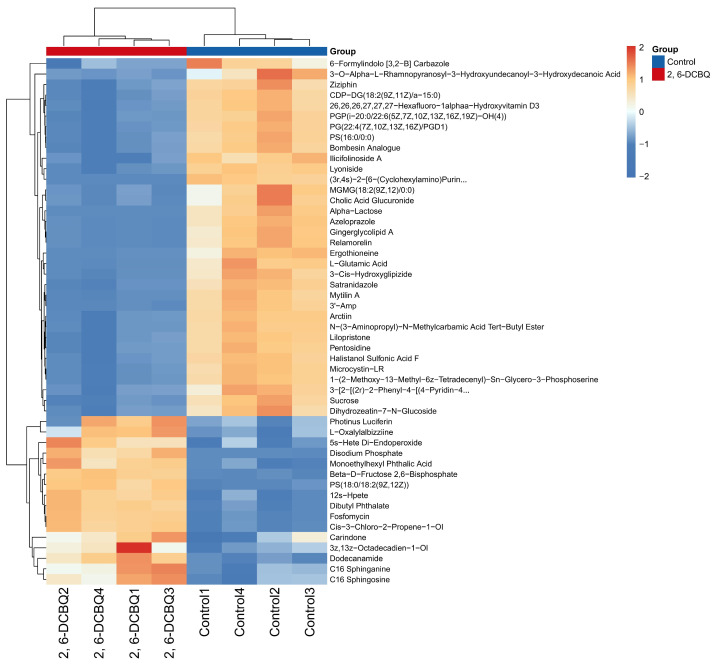
Cluster analysis of top 50 differential metabolites.

**Figure 7:**
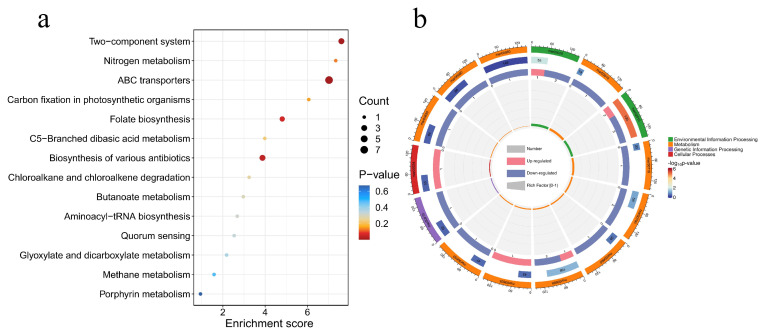
KEGG pathways with significant enrichment of metabolites (**a**). Circos diagram of KEGG pathways (**b**).

The volcano diagram in Figure 5c illustrates the number of significantly changed metabolites during the 0.1 mg/L 2,6-DCBQ treatment of *M. aeruginosa*. In addition, the volcano plot visually display the overall distribution of the DMs. A total of 63 upregulated (red dots) and 145 downregulated (blue dots) metabolites were identified during this period (Figure 5c). Figure 5d indicates the main differential metabolites; the box plots of relative abundance are shown in Figure S1. The upregulated DMs mainly comprised organic oxygen compounds and organic nitrogen compounds (Figure 6). The downregulated DMs primarily included lipids and lipid-like molecules, organic acids, and nucleotides (Figure 6). The key enriched pathways included ABC transporters, the two-component system, and folate biosynthesis (Figure 7a). Exposure to 2,6-DCBQ can affect the KEGG pathway of *M. aeruginosa* through environmental information processing, metabolism, genetic information processing, and cellular processes (Figure 7b). ABC transporters and the two-component system are involved in environmental information processing, while folate biosynthesis is a part of metabolism (Table S2). All differential metabolites showed both upregulation and downregulation. Quorum sensing is a part of cellular processes (Table S2), with all differential metabolites being upregulated. The differential metabolites of chloroalkane and chloroalkene degradation were all upregulated, while the differential metabolites of other metabolic pathways were all downregulated.

### 3.5. Gene Expressions

**Figure 8:**
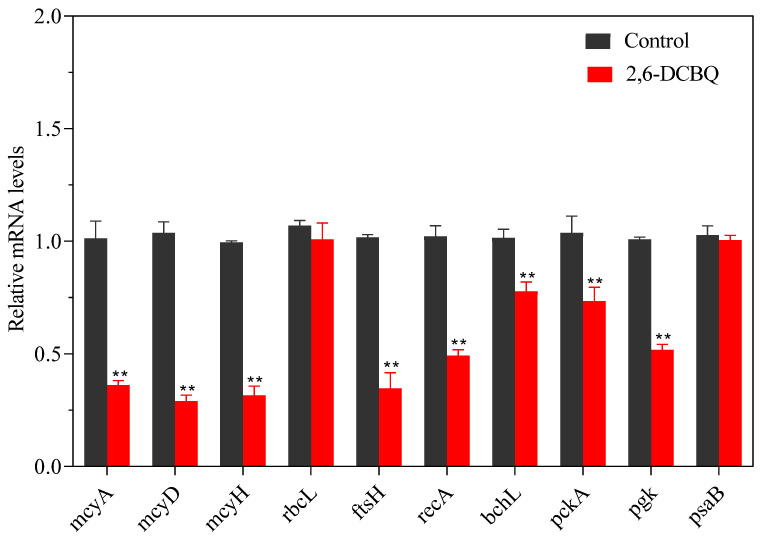
Gene expression levels in treatments with 0.1 mg/L 2,6-DCBQ after 3 days of exposure. Results are presented as means ± standard deviations. ** Indicates *p* < 0.01 relative to the control by ANOVA.

The expressions of genes involved in the energy metabolism, MCs, and photosynthesis were examined to explore the possible toxic mechanisms of 2,6-DCBQ. The results show that 2,6-DCBQ induced alterations in the gene expressions of *M. aeruginosa* (Figure 8). After the exposure of *M. aeruginosa* to 0.1 mg/L 2,6-DCBQ, the expressions of *mcyA*, *mcyD*, *mcyH*, *ftsH*, *recA*, *bchL*, *pckA*, and *pkg* revealed significant downregulation (*p* < 0.05). Meanwhile, the expressions of *rbcL* and *psaB* did not change significantly.

## 4. Discussion

### 4.1. Physiological Responses of M. aeruginosa Exposed to 2,6-DCBQ

#### 4.1.1. Growth and Photosynthetic Pigments

In this study, the results on cell density after exposure to 2,6-DCBQ indicate that 2,6-DCBQ exhibits acute toxicity (Figure 1a). *recA* plays a critical role in DNA repair [29,30]. The present study reveals that the significantly decreased expression of the *recA* gene suggests DNA damage may also indicate inhibited growth of *M. aeruginosa* due to exposure to 2,6-DCBQ (Figure 8).

The influence of 2,6-DCBQ on the chl-a and carotenoid contents was similar to those on cell growth. Furthermore, the decrease in the chl-a content could impair the antioxidant capability of *M. aeruginosa*, resulting in an energy deficit in the cells and reducing their resistance to ROS-induced damage [19]. Carotenoids can react with lipid peroxides and protect the photosynthetic system of *M. aeruginosa* [31,32]. Tiwari et al. [33] also noted that the heavy metal chromium can decrease the contents of chlorophyll and carotenoids in paddy-field cyanobacteria.

The decreases in chl-a (Figure 1b) suggest the impairment or disturbance of the photosynthetic system of *M. aeruginosa*, which hindered cell growth. Li et al. [34] confirmed the same phenomenon when they exposed *Chlorella pyrenoidosa* to perfluorooctanoic acid. In summary, 2,6-DCBQ restricted the expressions of photosynthesis-related genes in *M. aeruginosa*, which resulted in its decreased photosynthetic efficiency and inhibited growth. However, the result of the *psaB* gene suggests that photosystem I of *M. aeruginosa* was not severely damaged by 0.1 mg/L 2,6-DCBQ.

#### 4.1.2. ATP, Glucose, and Protein Contents

ATP is the direct energy source in organisms, driving various biochemical reactions and cellular activities. With prolonged exposure, the ATP content in the high-concentration treatment groups (0.1–0.5 mg/L 2,6-DCBQ) significantly increased, indicating that ATP is continuously synthesized to provide energy for repairing stress damage and other functions. A previous study also revealed that the chiral fragrance carvone affected the ATP production of *M. aeruginosa* [35], which is consistent with our experimental results.

The glucose and total soluble protein contents were comprehensively studied, and the results reveal the time–concentration toxic effect of 2,6-DCBQ on *M. aeruginosa*. In this study, reductions in the total soluble protein and glucose production were observed, indicating that disruptions in energy metabolism during photosynthesis may be involved in the growth inhibition of *M. aeruginosa* caused by 2,6-DCBQ.

The change in the total soluble protein content is a key response of *M. aeruginosa* under external stress. The total soluble protein content of M. aeruginosa decreased continuously under 2,6-DCBQ exposure (Figure 1d). The results indicate that the normal physiological activities of the cells were disrupted [35]. The *ftsH* gene plays a vital role in the maintenance of the quality of membrane proteins during the response to heat shock and in the regulation of cell division [36]. Midepogu et al. also found that after 96 h of treatment with TiO_2_ nanoparticles at a concentration of 20 mg/L, the *ftsH* gene in *Chlorella pyrenoidosa* decreased to approximately 0.3-fold of the control [37]. The downregulation of the cell division protein-related *ftsH* gene may be a direct cause of the inhibitory effect on cell growth (Figure 8).

#### 4.1.3. Oxidative Stress and Membrane Permeability

Excessive ROS production can affect various cellular processes through the alteration of nucleic acids, the oxidization of proteins, and the triggering of lipid peroxidation [38]. In this study, *M. aeruginosa* exposed to 2,6-DCBQ showed ROS accumulation and subsequent lipid peroxidation. In addition, excessive generation of ROS can initiate oxidative damage to the cell membrane, which results in morphological changes, such as disruption of cellular structures and cell death. To investigate the mechanism of oxidative damage, the effect of 2,6-DCBQ on lipid peroxidation was analyzed.

The results on the LPO content indicate that the lipids of *M. aeruginosa* were oxidatively damaged due to exposure to 2,6-DCBQ, and antioxidant enzymes cannot completely eliminate ROS [39]. In addition, a high concentration of 2,6-DCBQ may possibly induce the rise in LPO levels, which can increase cell membrane permeability [40]. The TEM results also confirm this conclusion (Figure 3b(2)). Our previous study further revealed that chloroacetic acid caused oxidative stress on *M. aeruginosa* [25].

Excessive production of SOD is the primary mechanism underlying the protection of thylakoid membranes against organic pollution [41]. Increased SOD activity protected green algae from the toxic effects of a pesticide known as trifloxystrobin [42]. The upregulation of CAT activity is considered an adaptation to the stress experienced by *M. aeruginosa*, which is stimulated by various environmental and chemical stressors [43].

#### 4.1.4. Production and Release of MC-LR

MCs are secondary metabolites of cyanobacteria [44]. Figure 4 indicates that 2,6-DCBQ increased the production and release of MC-LR. Exposure to emerging pollutants can upregulate the expressions of MC-related genes in *M. aeruginosa*, which will promote MC-LR secretion [45].

The *mcy* gene cluster plays a key role in the biosynthesis of MCs in various cyanobacterial genera, including *M. aeruginosa*. Genes *mcyA* and *mcyD* encode the synthetase of MCs. Gene *mcyH* encodes the transportation of MCs [46]. Numerous studies have verified the relationship between the upregulation of the relative transcript abundance of *mcy* gene and the increased contents of MC-LR during production [35,47]. In addition, the common contaminant γ-lindane upregulates the transcription of *mcyD* and *mcyH* genes and improves MC production in *M. aeruginosa* [48]. However, a previous study also reported that under external stressors, such as fluoroquinolone antibiotics, the extracellular concentration of MCs increased despite inhibited *M. aeruginosa* growth, potentially due to the regulation of the *mcy* cluster [49]. In the present study, genes of *mcyA*, *mcyD*, and *mcyH* were substantially decreased compared with the control (Figure 8). These results are possibly due to severe damage of the cell membrane (Figure 3b(4)), which may result in an increase in the extracellular MC-LR content.

### 4.2. Nontargeted Metabolomics Analysis

Such metabolites include substances related to antioxidation (alpha-lactose, sucrose, and dihydrozeatin-7-N-glucoside), as well as nucleosides, nucleotides and their analogues, organic acids, derivatives, and lipids, all of which were altered when *M. aeruginosa* was exposed to 2,6-DCBQ (Figure 6).

Glutamic acid can be transformed into alpha-ketoglutaric acid, which is an essential intermediate in the tricarboxylic acid (TCA) cycle, through the actions of glutamate dehydrogenase, alanine aminotransferase, or aspartate aminotransferase [32]. In photosynthetic organisms, the synthesis of chl starts from glutamate, which subsequently undergoes a series of intricate biochemical reactions that involve a minimum of 17 enzyme reaction steps [50]. The stress caused by 2,6-DCBQ resulted in downregulated contents of L-glutamic acid (Figure 6), which indicates that the TCA cycle was inhibited and amino acid metabolism was disrupted. In addition, the decline in genes encoding *pckA* (Figure 8) led to the blockage of the TCA cycle. Thus, 2,6-DCBQ can hinder the energy metabolism pathway.

Lipids are the main components of organelle membranes in cyanobacteria [51]. In this study, several fatty acids (such as the potassium salts of fatty acids) in the 2,6-DCBQ exposure group were downregulated, which indicates the decreased biosynthesis of some fatty acids. In addition, the fatty acid composition of *M. aeruginosa* changes under the stress of pollutants [28,52]. Glycerophospholipids take part in the development of biological membranes and contribute to protein recognition and cell membrane signaling [53]. In this study, numerous lipid DMs, such as dodecanamide, carindone, and gingerglycolipid, were screened based on the nontargeted metabolomics of 2,6-DCBQ-treated *M. aeruginosa*. This study hypothesized that 2,6-DCBQ would affect the lipid content, which would consequently alter the selective permeability of the biomembrane and lead to the death of *M. aeruginosa*. Consequently, alterations in the types and quantities of membrane-bound lipids result in the heightened permeability of the biomembrane system. Lipid metabolism is the core pathway for cell energy and substance metabolism; it also plays a key role in basic physiological processes, such as cell membrane formation and intracellular signal transduction. Changes in these pathways inevitably lead to disturbances across the entire metabolic network, consistent with findings in previous reports. Additionally, heatmap analysis revealed the relationship between metabolites and antioxidants (Figure 6).

The pathways significantly downregulated, including ABC transporters and the two-component system (*p* < 0.05) (Figure 7a). ABC transporters, particularly ABC transporter BmrD, can participate in the absorption, accumulation, and expulsion of endogenous toxins and exogenous substances, playing an important role in defense mechanisms [51,54]. The two-component system plays a crucial role in modulating responses to environmental changes. The results show that the related signaling pathways were inhibited. The downregulation of folate biosynthesis indicates that the folate cycle was inhibited. However, due to the high diversity of metabolism species and a limited pathway database, more DMs remain to be further studied in the future.

## 5. Conclusions

This work reveals the physiological responses and nontargeted metabolomics of *M. aeruginosa* exposed to various concentrations of 2,6-DCBQ. The possible mechanisms underlying the mortality of *M. aeruginosa* under 2,6-DCBQ stress can be summarized as follows: (1) 2,6-DCBQ hampered the synthesis of photosynthetic pigments and further influenced photosynthesis and energy metabolism; (2) 2,6-DCBQ increased ROS production and reduced the antioxidant capacity of *M. aeruginosa*; (3) 2,6-DCBQ affected the external morphology and cell structure of *M. aeruginosa* cells; (4) 2,6-DCBQ significantly increased the production and release of MC-LR while downregulating the expression of *mcyA*, *mcyD*, and *mcyH*, possibly due to the cell membrane damage; (5) the nontargeted metabolomics analysis identified 208 differential metabolites (including antioxidants, substances related to energy metabolism, and lipids) belonging to 10 metabolic pathways. Significant metabolic enrichment pathways include ABC transporters, two-component systems, and folate biosynthesis. This study revealed the inhibitory mechanism and key biomarkers of 2,6-DCBQ on *M. aeruginosa* through in situ bioanalytical methods with multiple endpoints and nontargeted metabolomics analyses. This study also provides a theoretical basis for future research on risk assessments and the development of water quality criteria for new pollutants such as DBPs.

## Data Availability

Data will be made available upon request.

## Supplementary Materials

The following supporting information can be downloaded at https://www.mdpi.com/article/10.3390/toxics13010064/s1: Figure S1: Box plots of relative abundance of C16, Arctiin, Gingerglycolipid A, Microcystin-Lr, Halistanol Sulfonic Acid F, Lilopristone, 1-(2-Methoxy-13-Methyl-6z-Tetradecenyl)-Sn-Glycero-, MGMG(18:2(9Z,12)/0:0), Mytilin A, PS(16:0/0:0), Photinus Luciferin, Relamorelin, Disodium Phosphate, Beta-D-Fructose 2,6-Bisphosphate, Fosfomycin, L-Oxalylalbizziine, Cis-3-Chloro-2-Propene-1-Ol, C16 Sphingosine, Dibutyl Phthalate, and Dodecanamide; Table S1: PCR primer sequences used in the experiment; Table S2: Detailed information of Circos diagram.
