# Supplementary material for: The Effects of Disinfection Byproduct 2,6-Dichloro-1,4-benzoquinone on the Cyanobacterium Microcystis aeruginosa: From the Perspectives of Biochemistry and Non-Targeted Metabolomics
Source: Toxics. 2025 Jan 17;13(1):64. doi: 10.3390/toxics13010064 (PMC11768523; doi:10.3390/toxics13010064)
Supplement: Supplementary file 1 [file toxics-13-00064-s001.zip › toxics-3389367-supplementary.pdf]

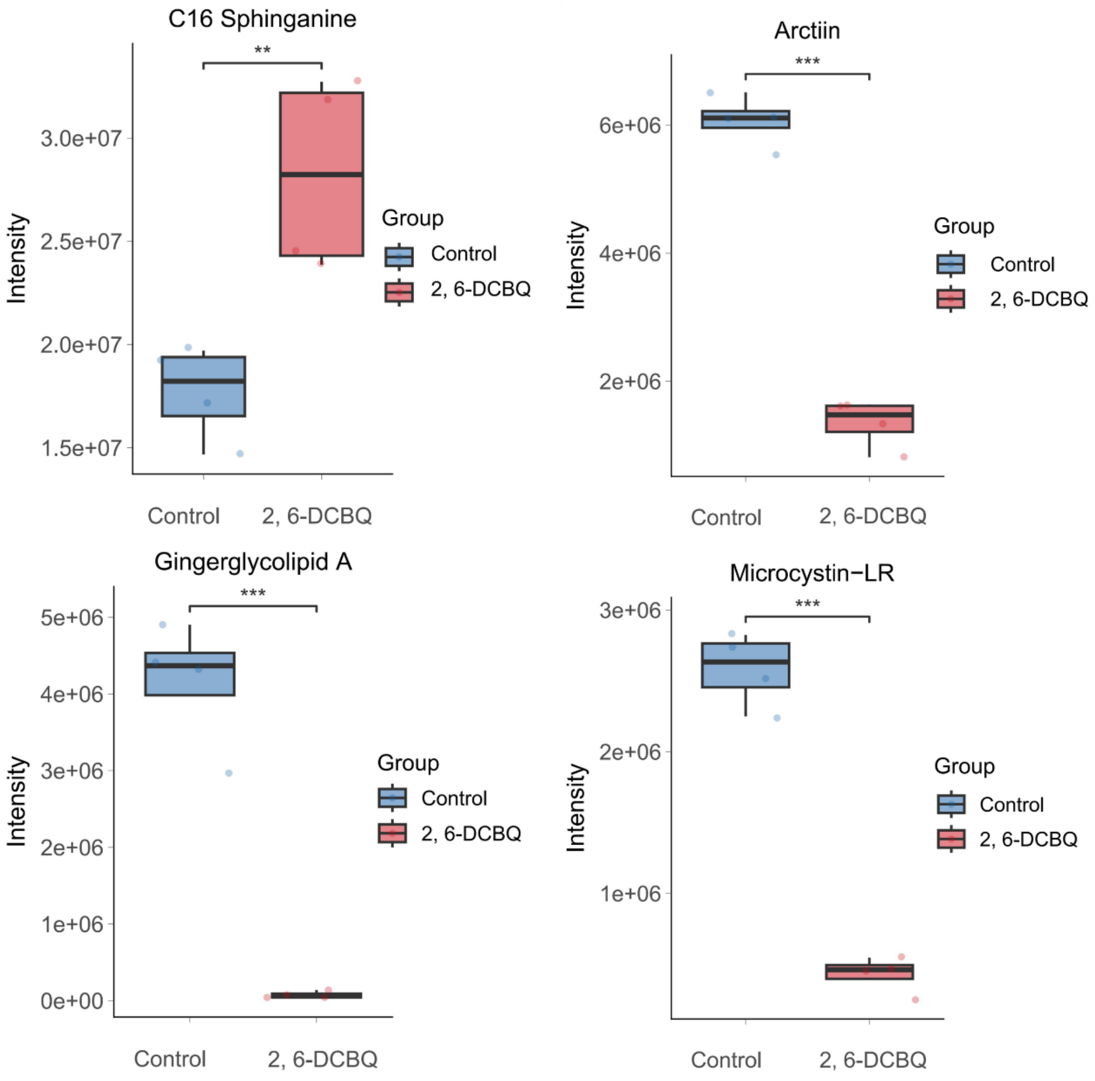
C16 Sphinganine
**
3.0e+07
2.5e+07
2.0e+07
1.5e+07
Intensity
Control
2, 6-DCBQ
Group
Control
2, 6-DCBQ
Arctiin
***
6e+06
4e+06
2e+06
Intensity
Control
2, 6-DCBQ
Group
Control
2, 6-DCBQ
Gingerglycolipid A
***
5e+06
4e+06
3e+06
2e+06
1e+06
0e+00
Intensity
Control
2, 6-DCBQ
Group
Control
2, 6-DCBQ
Microcystin−LR
***
3e+06
2e+06
1e+06
Intensity
Control
2, 6-DCBQ
Group
Control
2, 6-DCBQ

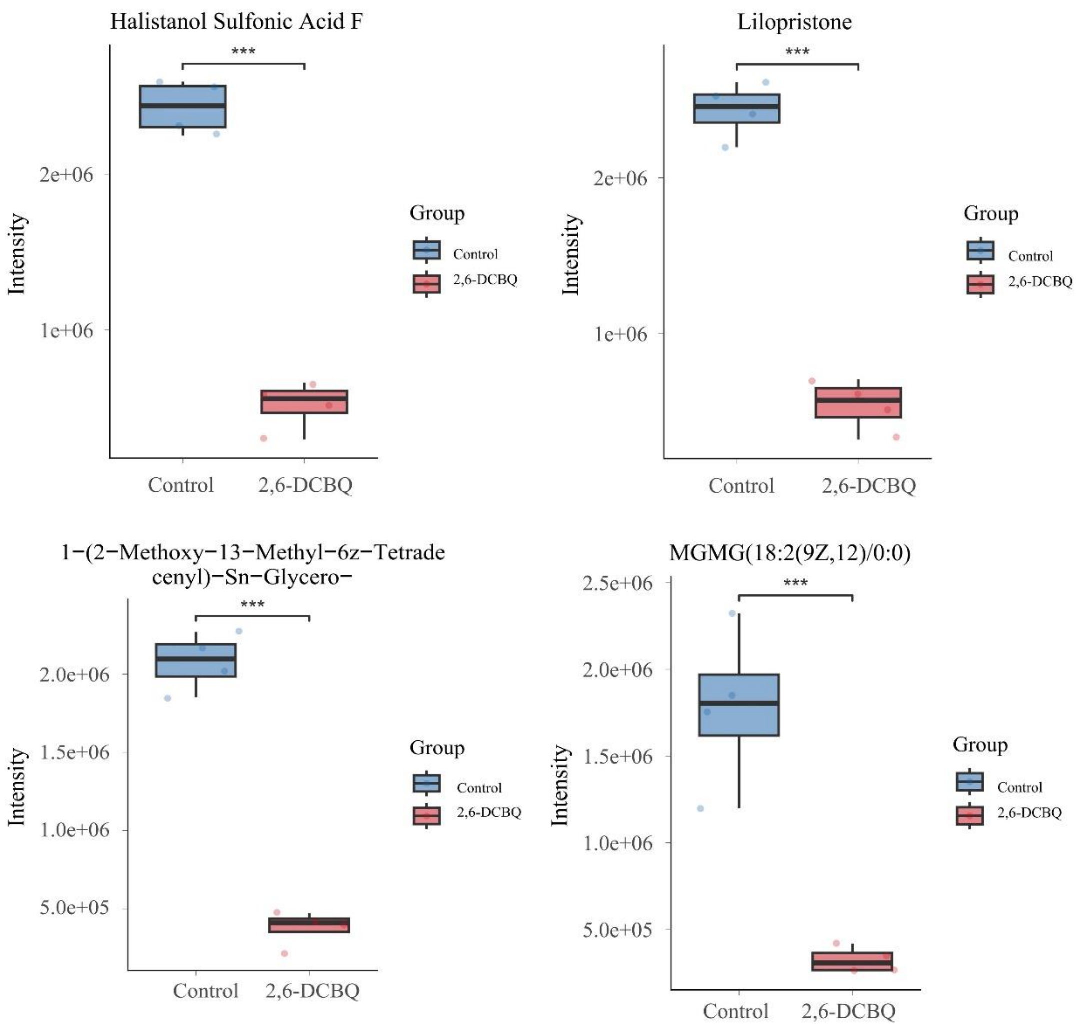
Halistanol Sulfonic Acid F
***
2e+06
1e+06
Intensity
Control
2,6-DCBQ
Group
Control
2,6-DCBQ
Lilopristone
***
2e+06
1e+06
Intensity
Control
2,6-DCBQ
Group
Control
2,6-DCBQ
1−(2−Methoxy−13−Methyl−6z−Tetrade
cenyl)−Sn−Glycero−
***
2.0e+06
1.5e+06
1.0e+06
5.0e+05
Intensity
Control
2,6-DCBQ
Group
Control
2,6-DCBQ
MGMG(18:2(9Z,12)/0:0)
***
2.5e+06
2.0e+06
1.5e+06
1.0e+06
5.0e+05
Intensity
Control
2,6-DCBQ
Group
Control
2,6-DCBQ

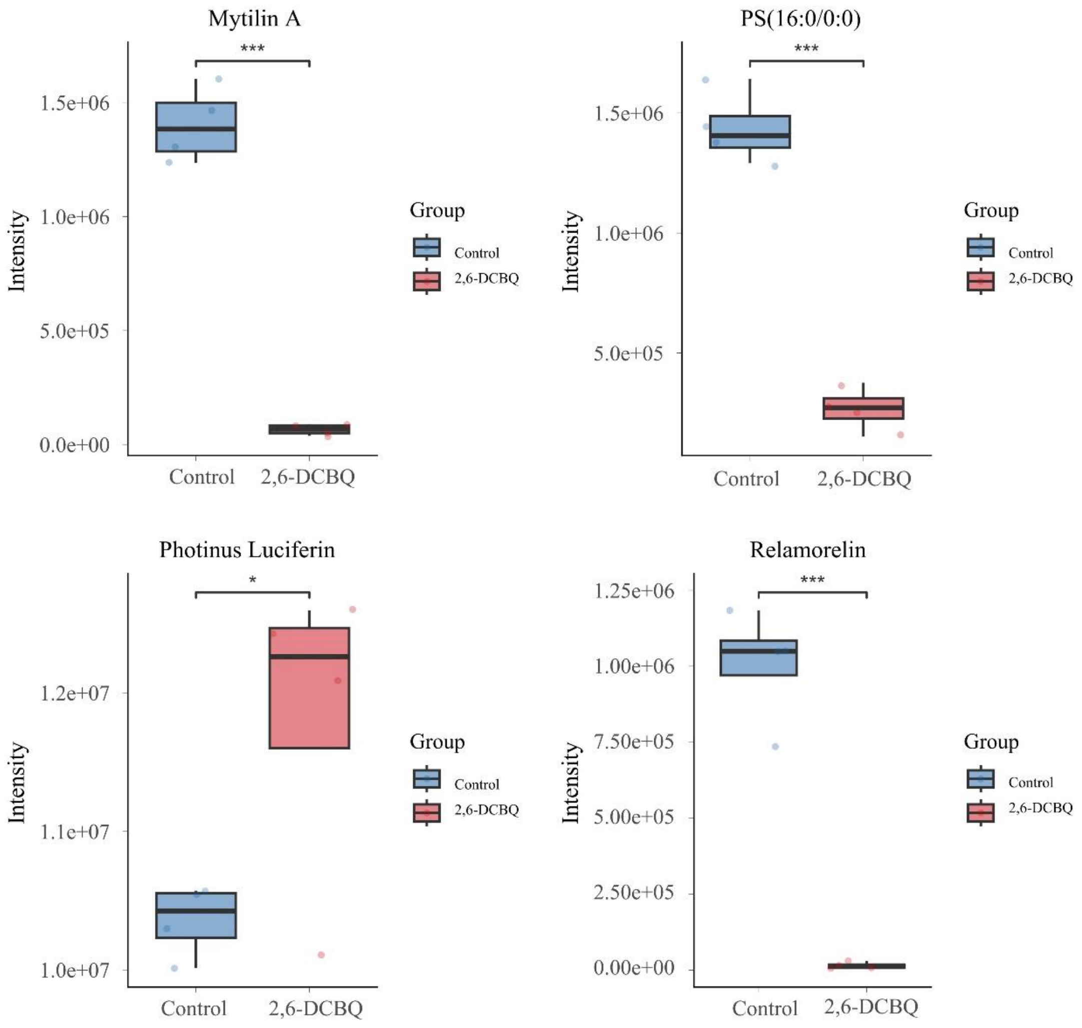

Mytilin A
***
1.5e+06
1.0e+06
5.0e+05
0.0e+00
Intensity
Control
2,6-DCBQ
Group
Control
2,6-DCBQ
PS(16:0/0:0)
***
1.5e+06
1.0e+06
5.0e+05
Intensity
Control
2,6-DCBQ
Group
Control
2,6-DCBQ
Photinus Luciferin
*
1.2e+07
1.1e+07
1.0e+07
Intensity
Control
2,6-DCBQ
Group
Control
2,6-DCBQ
Relamorelin
***
1.25e+06
1.00e+06
7.50e+05
5.00e+05
2.50e+05
0.00e+00
Intensity
Control
2,6-DCBQ
Group
Control
2,6-DCBQ

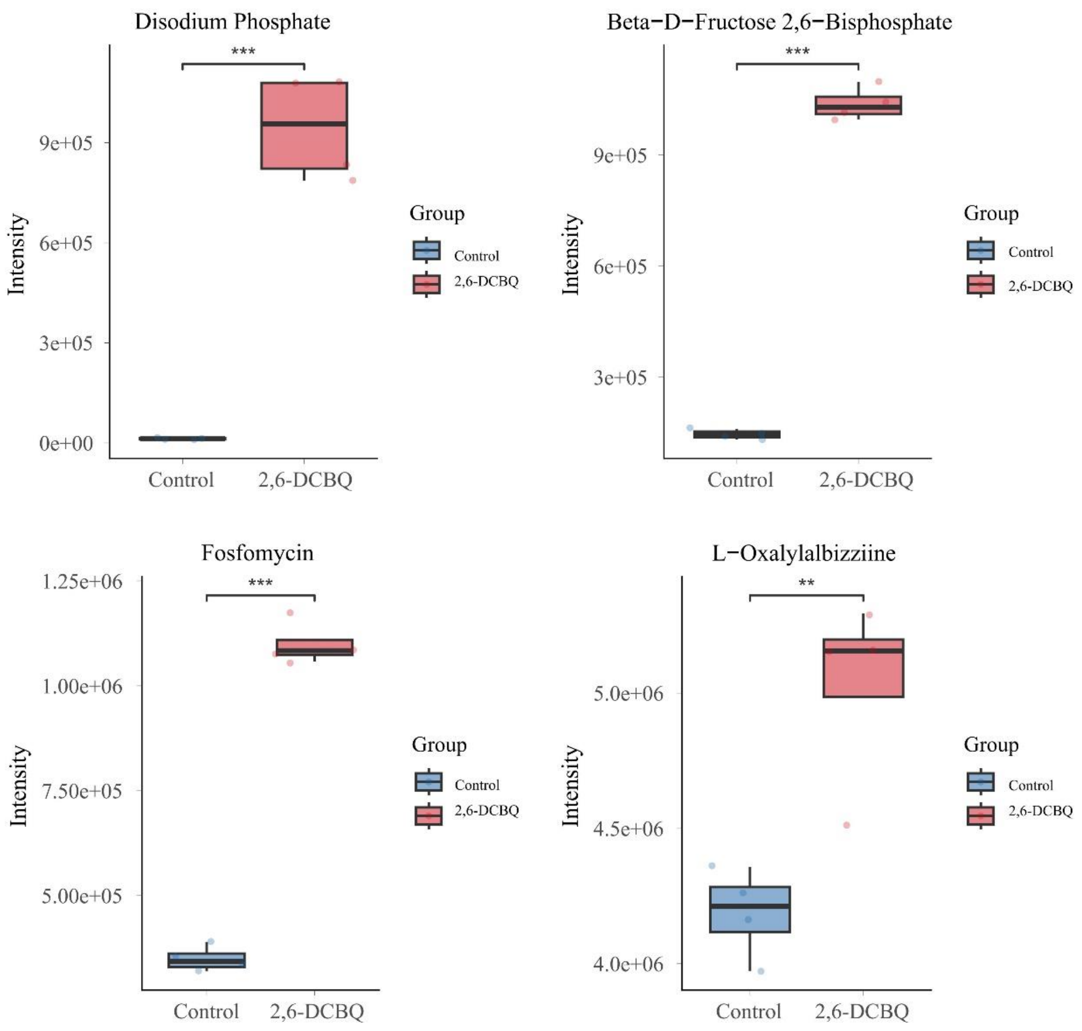
Disodium Phosphate
***
Intensity
9e+05
6e+05
3e+05
0e+00
Control
2,6-DCBQ
Group
Control
2,6-DCBQ
Beta−D−Fructose 2,6−Bisphosphate
***
Intensity
9e+05
6e+05
3e+05
Control
2,6-DCBQ
Group
Control
2,6-DCBQ
Fosfomycin
***
Intensity
1.25e+06
1.00e+06
7.50e+05
5.00e+05
Control
2,6-DCBQ
Group
Control
2,6-DCBQ
L−Oxalylalbizziine
**
Intensity
5.0e+06
4.5e+06
4.0e+06
Control
2,6-DCBQ
Group
Control
2,6-DCBQ

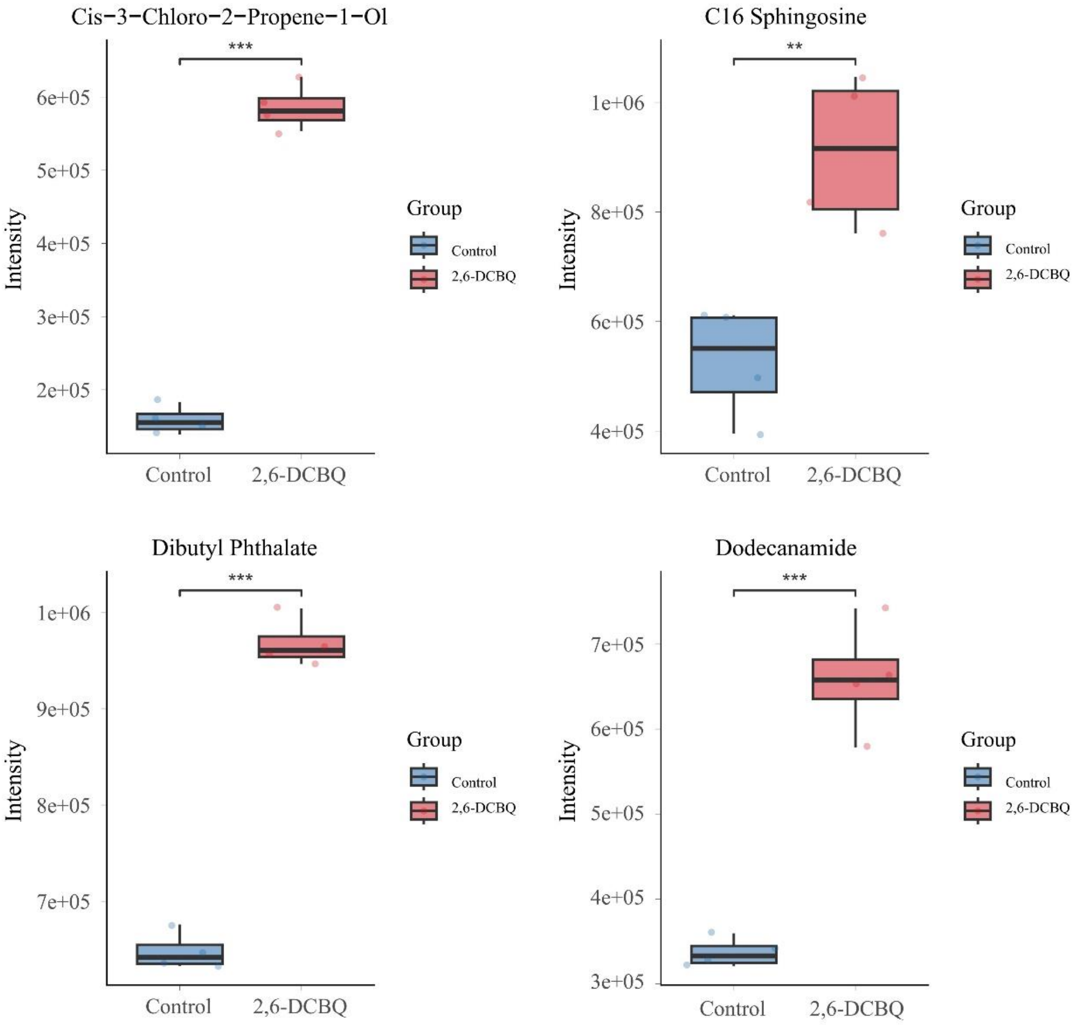

Figure S1. Box plots of relative abundance of C16, Arctiin, Gingerglycolipid A, Microcystin-LR, Halistanol Sulfonic Acid F, Lilopristone, Lilopristone, 1-(2-Methoxy-13-Methyl-6z-Tetradecenyl)-Sn-Glycero-, MGMG(18:2(9Z,12)/0:0), Mytilin A, PS(16:0/0:0), Photinus Luciferin, Relamorelin, Disodium Phosphate, Beta-D-Fructose 2,6-Bisphosphate, Fosfomycin, L-Oxalylalbizziine, Cis-3-Chloro-2-Propene-1-Ol, C16 Sphingosine, Dibutyl Phthalate, Dodecanamide.

Table S1. PCR primer sequences used in the experiment.

Gene name	Primer sequence	Biomarker
16S rRNA	FORWARD GGACGGGTGAGTAACGCGTA<br>REVERSE CCCATTGCGGAAAATTCCCC	/
psaB	FORWARD CGGTGACTGGGGTGTGTATG<br>REVERSE ACTCGGTTTGGGGATGGA	photosystem I
ftsH	FORWARD CCTCATTGACGAGGTTGGCT<br>REVERSE TGGATCGTCCCGATTTAGCG	cell division
rbcL	FORWARD TCGGTGACGATTCCTGCTT<br>REVERSE AGGACTCCAACGGCAAGC	photosystem II
mcyA	FORWARD GCCGATGTTTGGCTGTAAAT<br>REVERSE ATCCAGCAGTTGACCAAGC	synthesis of microcystin
mcyD	FORWARD TTACCCCTTTCGGCTCGTTC<br>REVERSE GAAAAATCCCCCGCCCTGTA	synthesis of microcystin
mcyH	FORWARD ACAACCCGTGAAATGACCGA<br>REVERSE TCCCAAGGAACTTCCGCATC	transport of microcystin
psbB	FORWARD CGGTGACTGGGGTGTGTATG<br>REVERSE ACTCGGTTTGGGGATGGA	photosystem II
bchL	FORWARD TCGGTGACGATTCCTGCTT<br>REVERSE AGGACTCCAACGGCAAGC	negative regulatory factors of cell death
pgk	FORWARD CAATAGTTGGTAGTGCCGCC<br>REVERSE ACAGAGGCTGATTTAGCGGG	glycolysis
pckA	FORWARD TGCCGAATACGAACCCCAAA<br>REVERSE CGTCCATCATCGTAGTCGGG	TCA cycle

Table S2. Detailed information of circos diagram.

Id	Classification level	Term
mar02010	Environmental Information Processing	ABC transporters
mar02020	Environmental Information Processing	Two-component system
mar00998	Metabolism	Biosynthesis of various antibiotics
mar00790	Metabolism	Folate biosynthesis
mar00910	Metabolism	Nitrogen metabolism
mar00710	Metabolism	Carbon fixation in photosynthetic organisms
mar00660	Metabolism	C5-Branched dibasic acid metabolism
mar00625	Metabolism	Chloroalkane and chloroalkene degradation
mar00650	Metabolism	Butanoate metabolism
mar00970	Genetic Information Processing	Aminoacyl-tRNA biosynthesis
mar02024	Cellular Processes	Quorum sensing
mar00630	Metabolism	Glyoxylate and dicarboxylate metabolism
mar00680	Metabolism	Methane metabolism
mar00860	Metabolism	Porphyrin metabolism
